# Private sector role, readiness and performance for malaria case management in Uganda, 2015

**DOI:** 10.1186/s12936-017-1824-x

**Published:** 2017-05-25

**Authors:** ACTwatch Group, Louis Akulayi, Angela Alum, Andrew Andrada, Julie Archer, Ekundayo D. Arogundade, Erick Auko, Abdul R. Badru, Katie Bates, Paul Bouanchaud, Meghan Bruce, Katia Bruxvoort, Peter Buyungo, Angela Camilleri, Emily D. Carter, Steven Chapman, Nikki Charman, Desmond Chavasse, Robyn Cyr, Kevin Duff, Gylsain Guedegbe, Keith Esch, Illah Evance, Anna Fulton, Hellen Gataaka, Tarryn Haslam, Emily Harris, Christine Hong, Catharine Hurley, Whitney Isenhower, Enid Kaabunga, Baraka D. Kaaya, Esther Kabui, Beth Kangwana, Lason Kapata, Henry Kaula, Gloria Kigo, Irene Kyomuhangi, Aliza Lailari, Sandra LeFevre, Megan Littrell, Greta Martin, Daniel Michael, Erik Monroe, Godefroid Mpanya, Felton Mpasela, Felix Mulama, Anne Musuva, Julius Ngigi, Edward Ngoma, Marjorie Norman, Bernard Nyauchi, Kathryn A. O’Connell, Carolyne Ochieng, Edna Ogada, Linda Ongwenyi, Ricki Orford, Saysana Phanalasy, Stephen Poyer, Justin Rahariniaina, Jacky Raharinjatovo, Lanto Razafindralambo, Solofo Razakamiadana, Christina Riley, John Rodgers, Andria Rusk, Tanya Shewchuk, Simon Sensalire, Julianna Smith, Phok Sochea, Tsione Solomon, Raymond Sudoi, Martine Esther Tassiba, Katherine Thanel, Rachel Thompson, Mitsuru Toda, Chinazo Ujuju, Marie-Alix Valensi, Vamsi Vasireddy, Cynthia B. Whitman, Cyprien Zinsou, Henry Kaula, Peter Buyungo, Jimmy Opigo

**Affiliations:** 1 Population Services International, 1120 19th St NW Suite 600, Washington, DC 20036 USA; 2 Programme for Accessible Communication and Education (PACE) Uganda, Plot # 2, Ibis Vale, Kololo-off Prince Charles Drive, Kampala, Uganda; 3 National Malaria Control Programme, Ministry of Health, Kampala, Uganda

**Keywords:** Private sector, Case management, Anti-malarial, ACT, Diagnostics, RDT

## Abstract

**Background:**

Several interventions have been put in place to promote access to quality malaria case management services in Uganda’s private sector, where most people seek treatment. This paper describes evidence using a mixed-method approach to examine the role, readiness and performance of private providers at a national level in Uganda. These data will be useful to inform strategies and policies for improving malaria case management in the private sector.

**Methods:**

The ACTwatch national anti-malarial outlet survey was conducted concurrently with a fever case management study. The ACTwatch nationally representative anti-malarial outlet survey was conducted in Uganda between May 18th 2015 and July 2nd 2015. A representative sample of sub-counties was selected in 14 urban and 13 rural clusters with probability proportional to size and a census approach was used to identify outlets. Outlets eligible for the survey met at least one of three criteria: (1) one or more anti-malarials were in stock on the day of the survey; (2) one or more anti-malarials were in stock in the 3 months preceding the survey; and/or (3) malaria blood testing (microscopy or RDT) was available. The fever case management study included observations of provider-patient interactions and patient exit interviews. Data were collected between May 20th and August 3rd, 2015. The fever case management study was implemented in the private sector. Potential outlets were identified during the main outlet survey and included in this sub-sample if they had both artemisinin-based combination therapy (ACT) [artemether–lumefantrine (AL)], in stock on the day of survey as well as diagnostic testing available.

**Results:**

A total of 9438 outlets were screened for eligibility in the ACTwatch outlet survey and 4328 outlets were found to be stocking anti-malarials and were interviewed. A total of 9330 patients were screened for the fever case management study and 1273 had a complete patient observation and exit interview. Results from the outlet survey illustrate that the majority of anti-malarials were distributed through the private sector (54.3%), with 31.4% of all anti-malarials distributed through drug stores and 14.4% through private for-profit health facilities. Availability of different anti-malarials and diagnostic testing in the private sector was: ACT (80.7%), quality-assured (QA) ACT (72.0%), sulfadoxine–pyrimethamine (SP) (47.1%), quinine (73.2%) and any malaria blood testing (32.9%). Adult QAACT ($1.62) was three times more expensive than SP ($0.48). The results from the fever case management study found 44.4% of respondents received a malaria test, and among those who tested positive for malaria, 60.0% received an ACT, 48.5% received QAACT; 14.4% a non-artemisinin therapy; 14.9% artemether injection, and 42.5% received an antibiotic.

**Conclusion:**

The private sector plays an important role in malaria case management in Uganda. While several private sector initiatives have improved availability of QAACT, there are gaps in malaria diagnosis and distribution of non-artemisinin monotherapies persists. Further private sector strategies, including those focusing on drug stores, are needed to increase coverage of parasitological testing and removal of non-artemisinin therapies from the marketplace.

**Electronic supplementary material:**

The online version of this article (doi:10.1186/s12936-017-1824-x) contains supplementary material, which is available to authorized users.

## Background

The private sector is an important provider of health services in Uganda, with up to 80% of patients seeking treatment from this sector [1, 2]. Private health facilities in Uganda have included private for-profit hospitals and clinics, pharmacies and drug stores—the latter which can be licensed and unlicensed private sector outlets [3]. In particular, drug stores, which constitute a large proportion of Uganda’s private sector have been found to be one of the first points of care with an estimated 50% of all anti-malarials distributed through these outlets [2, 4].

Since 2004, the Ugandan anti-malarial treatment policy has stipulated the use of artemisinin-based combination therapy (ACT) for uncomplicated malaria. Prior to 2010, these treatment guidelines advocated for presumptive treatment of all suspected malaria cases, and it was stipulated that even confirmed negative cases were to be administered ACT [5]. The goal of this blanket policy was to reduce the risk of severe illness or death as a result of malaria [6]. However, since 2010 the guidelines have been updated to specify that prior to treatment, all cases of suspected malaria should receive a malaria blood test, and only patients testing positive for malaria should be administered an ACT [7].

These policy changes have been complemented by several private sector initiatives to ensure patients are tested and treated according the national malaria treatment guidelines. Uganda’s current policy stipulates that licensed private sector outlets are authorized to sell over-the-counter medicines including anti-malarials (and ACT as of 2008) but not antibiotics or injections [8]. Diagnostic testing with rapid diagnostic tests (RDTs) is only permitted in approved pilot areas of the country.

In 2010, Uganda participated in the Affordable Medicines Facility-malaria (AMFm) with the aim of increasing uptake of quality-assured ACT (QAACT) and decreasing use of artemisinin monotherapies. The programme was designed as a ‘factory-gate’ subsidy, reducing the cost of ACT to public and private sector first-line buyers by roughly 95% [9]. Following the AMFm pilot phase from 2010 to 2011, the programme of subsidies and price negotiations continued as part of Uganda’s malaria funding application to the Global Fund, and was called the Private Sector Co-payment Mechanism (CPM) for QAACT. Several supporting interventions in Uganda planned for implementation for the subsidy programme included behaviour change communications (BCC), the training of private sector providers, and the introduction of recommended retail prices for QAACT; however, there were challenges which prevented the BCC activities from being implemented [10]. All subsidized QAACT packaging carried a green leaf logo as an indication of quality and affordable anti-malarial treatment. The Independent Evaluation of the AMFm concluded that overall there was a significant increase in availability of QAACT in the private sector following AMFm implementation, from 11.3% in 2010 to 65.5% in 2011 [11].

Aside from the CPM, more recent examples of strategies to improve malaria case management services include the introduction of malaria diagnostics into licensed private outlets, typically drug shops [12–14]. These pilot interventions have included the provision of subsidized RDTs and have been implemented with supportive interventions, including training and supervision of providers. Several studies have concluded that RDTs can be stocked and used safely to treat malaria outside formal health facilities in Uganda [15, 16] and that their use can lead to reduced prescription of anti-malarial drugs among RDT negative patients [12, 13, 17]. Given these positive findings, the policy on diagnostic testing in the private sector is under review by the government.

The various private sector investments discussed above have played an important role in improving private sector malaria case management readiness and performance in Uganda. Contemporary malaria case management market data on anti-malarials and malaria diagnostics will provide an important benchmark of this success. Since 2008, the ACTwatch project has been implemented in Uganda to monitor anti-malarial and diagnostic markets. To date, five national outlet surveys have been implemented across the country. This paper describes evidence from Uganda’s last survey round implemented in 2015 and examines the role, readiness and performance of private providers at a national level in Uganda. It is complemented with a fever case management survey to explore the performance of private sector and adherence to national guidelines by private providers. These data will be useful to inform and improve strategies and policies for malaria case management in Uganda’s private sector.

## Methods

The ACTwatch national anti-malarial outlet survey was conducted concurrently with the fever case management study, but they differed in their design and sampling approaches.

### Outlet survey

The ACTwatch nationally representative anti-malarial outlet survey was conducted in Uganda between May 18th 2015 and July 2nd 2015. A representative sample of 27 sub-counties was selected in urban (14) and rural (13) domains with probability proportional to size. Within selected clusters, a census of all outlets with the potential to sell or distribute anti-malarials and/or provide malaria blood testing was completed. In Uganda, these outlet types included public health facilities, community health workers (CHW), private not-for-profit health facilities, private for-profit health facilities, pharmacies, and drug stores. Additional sub-counties were selected for oversampling of public health facilities and pharmacies. This booster sampling strategy was used to obtain a sufficient sample size for indicator estimates within these important outlet types.

The outlet survey was powered to detect a minimum of a 20% point change in availability of QAACT among anti-malarial stocking outlets between each round and within each domain at the 5% significance level with 80% power. The number of survey clusters was calculated for each research domain based on the required number of anti-malarial stocking outlets and assumptions about the number of anti-malarial stocking outlets per cluster. Sample size requirements for the 2015 survey were calculated using information from the previous survey round including anti-malarial and QAACT availability, outlet density per cluster, and design effect.

To implement the census, interviewers moved systematically through each of the selected clusters, looking for the aforementioned outlets. Where available, lists of registered licensed outlets were used to help identify any outlets. Snowball sampling was also used by interviewers to make sure all potential outlets were identified during the census process. Maps, illustrating local boundaries, were also used to identify the administrative boundaries of each cluster.

Outlets were screened to determine eligibility. Outlets eligible for the survey met at least one of three criteria: (1) one or more anti-malarials were in stock on the day of the survey; (2) one or more anti-malarials were in stock in the 3 months preceding the survey; and/or (3) malaria blood testing (microscopy or RDT) was available.

Among outlets that met the criteria, the main questionnaire with a malaria and RDT audit sheet was administered to consenting providers. Providers were asked to show the interviewer all anti-malarials currently available. A product audit sheet captured information for each unique anti-malarial product in the outlet, including formulation, brand name, active ingredients and strengths, package size, manufacturer and country of manufacture. Providers were asked to report the retail and wholesale price for each medicine as well as the amount distributed to individual consumers in the last week.

Quality control measures implemented during data collection included questionnaire review by supervisors and interview verification visits conducted by quality controllers among 10 and 20% of all outlets.

### Fever case management study

The fever case management study employed a cross-sectional quantitative design, including observations of provider-patient interactions and patient exit interviews. Data were collected between May 20th and August 3rd, 2015.

The fever case management study was implemented in the private sector, among private for-profit health facilities, pharmacies and drug stores. Potential outlets were identified during the main outlet survey and included in this sub-sample if they had both the first-line treatment ACT [artemether–lumefantrine (AL)], in stock on the day of survey as well as diagnostic testing available. Observation and exit interviews were conducted within a few days of completion for the the main outlet survey.

The target population for the fever case management study included providers and patients, or their caregivers, seeking fever treatment. The inclusion criteria were: patients (or their caregivers) with fever or history of fever, seeking care at the outlet for this fever for the first time; minimum 18 years of age (or 2 months of age providing the caregiver was at least 18 years of age); not currently pregnant; and not experiencing symptoms of severe illness.

Among eligible outlets with consenting providers, patients or their caregivers seeking treatment for fever were sampled for inclusion in the study. All patients meeting eligibility criteria as outlined above were invited to participate in the study. A quota sampling approach was used, with the aim of achieving two interviews per outlet: one from a caregiver on behalf of a child under the age of five and one from an adult/or the caregiver of a patient over the age of five. Following informed consent procedures, a structured observation checklist was completed by an interviewer observing the interactions that the patient had with providers as she/he was provided with services at the outlet. The observation was concerned primarily with provider behaviors, including the provider assessment of the patient, the administration of RDT and counseling for treatment with ACT. A brief exit interview was completed with the patient when he or she left the outlet. The exit interview was concerned with capturing information about all medicines prescribed/obtained. The exit interview also assessed the patient understanding of the test result(s) and medication regimens prescribed. Once the quota of two interviews per outlet was achieved, the interviewers moved on to the next eligible outlet. A maximum of 1 day was spent at the outlet by interviewers, and if the patient quota was not met, the interviewers moved to the next outlet.

### Training

Interviewers, supervisors, and quality controllers received training that included an orientation to the study designs and questionnaires, classroom training on completing observation and exit interviews, and a practice field exercise. Additional training was provided for supervisors and quality-controllers focused on field monitoring, verification visits, and census procedures.

### Protection of human subjects

Both the main outlet survey and the fever case management study were submitted for ethical review. The application was reviewed and approved by the Makerere University College of Health Sciences School of Medicine Research Ethics Committee (REC REF No. 2008-057). The PSI Research Ethics Board ceded review to the ethics committee in Uganda. Provider interviews, patient consultation observation, and patient exit interviews were completed only after administration of a standard informed consent form and patient/provider consent to participate in the study. Patients and providers had the option to end the interview at any point during the study. Standard measures were employed to maintain confidentiality and anonymity.

### Data entry

Different approaches were used for the main outlet survey and the fever case management study. A structured questionnaire programmed into mobile phones using DroidDB software was used to complete an audit of all anti-malarials and RDTs as well as a provider interview for the main outlet survey. Paper questionnaires were used to collect data for the fever case management study. A Microsoft Access (©Microsoft, Redmond, WA) database was developed and used to conduct double data entry from fever case management questionnaires. Verification records from data entry and supervisor monitoring sheets were reviewed and used to confirm complete data entry.

### Analysis

Stata 13.1 (©StataCorp, College Station, TX) was used to clean and analyse data from the outlet survey and fever case management study. Sampling weights were applied to account for variations in probability of selection and standard error estimation accounted for clustering at the sub-district level for the outlet survey. All point estimates were weighted using survey settings and all standard errors calculated taking account of the clustered and stratified sampling strategy with the relevant suite of survey commands.

For the outlet survey, standard indicators were constructed according to definitions applied across the ACTwatch project and have been described in detail elsewhere [18, 19]. Briefly, anti-malarials identified during the outlet drug audit were classified according to information on drug formulation, active ingredients and strengths as non-artemisinin therapies, artemisinin monotherapies and ACT. Non-artemisinin therapies were classified as sulfadoxine–pyrimethamine (SP), or other non-artemisinin therapies. Artemisinin monotherapies were further classified as oral and non-oral, the latter including medicines recommended for the first-line treatment of severe malaria. ACT were classified as QAACT or non QAACT. QAACT were either ACT products granted World Health Organization (WHO) prequalification, those granted regulatory approval by the European Medicines Agency (EMA) or those in compliance with the Global Fund Quality Assurance Policy. Classification was completed by matching product audit information (formulation, active ingredients, strengths, manufacturer, country of manufacturer and package size) to the most recent lists of approved medicines available from the WHO, EMA and Global Fund.

Anti-malarial availability and malaria diagnostic availability is presented out of all screened outlets in the private sector and by outlet type.

To calculate market share, anti-malarial sales were standardized to allow meaningful comparisons between anti-malarials with different treatment courses and different formulations. The adult equivalent treatment dose (AETD) was defined as the amount of active ingredient required to treat an adult weighing 60 kg according to WHO treatment guidelines [7]. Provider reports on the amount of the drug sold or distributed during the week preceding the survey were used to calculate volumes in AETDs according to type of anti-malarial. Measures of volume include all dosage forms to provide a complete assessment of anti-malarial market share. Market share is presented within the private sector and within each private sector outlet type.

Price data presented were collected in Ugandan shilling and converted to United States dollars using local exchange rates for the period of data collection. The price of QAACT was presented as the price of pre-packaged therapy for a 60 kg adult (i.e. AL 20/120, package size of 24 tablets), and the price of pre-packaged therapy for a 10 kg child (i.e. AL 20/120 package size of 6 tablets). Median private sector price per AETD was also calculated for QAACT and for the most popular non-artemisinin therapy in the most recent round, SP. The interquartile range (IQR) is presented as a measure of dispersion. While all QAACT are by definition tablet formulations, SP may be available in other formulations including syrups and injections. Price measures for QAACT, SP, adult QA AL and child QA AL included tablet anti-malarials only, given differences in unit costs for tablet and non-tablet formulations. Price was also calculated for an ampule of quinine and artemether injection, and presented separately.

The private sector price of a malaria test using microscopy or RDT was assessed through provider reports of consumer prices. Providers were asked to report the total cost of testing to a customer including any consultation or service fees. Median private sector price for microscopy or RDTs was calculated and reported with the IQR as a measure of dispersion.

The fever case management indicators include respondents that completed both the observation and exit interview components. Indicators include a description of the sample, including whether or not the febrile patient was present at the consultation and if the respondent had sought treatment elsewhere. Point estimates were also calculated to present data on whether or not the respondent received a test, the type of test received, and the result of the test (tested positive, tested negative, not tested). The types of medicines received were classified according to anti-malarials as well as antipyretics, and antibiotics.

## Results

### Outlet survey

A total of 9438 outlets were screened for availability of anti-malarials and/or malaria blood testing services. Of screened outlets, 4598 were stocking anti-malarials or testing on the day of the survey or within the past 3 months, and 4724 were subsequently interviewed (Additional file 1: Table S1).

### Anti-malarial market share

**Fig. 1:**
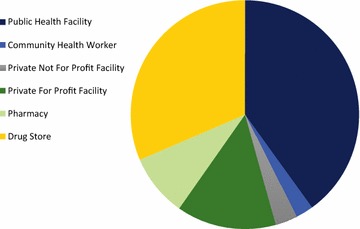
Anti-malarial market share

Figure 1 illustrates the market share of anti-malarials distributed according to different outlet types and by sector. The majority of the anti-malarials were distributed through the private sector (54.3%), with third of all anti-malarials distributed through drug stores (31.4%), followed by private for-profit health facilities (14.1%) and pharmacies (8.8%). In comparison, 45.7% of the anti-malarial market share was distributed through the public sector, with most anti-malarials being administered through public health facilities (40.0%).

### Anti-malarial market share within the private sector

**Fig. 2:**
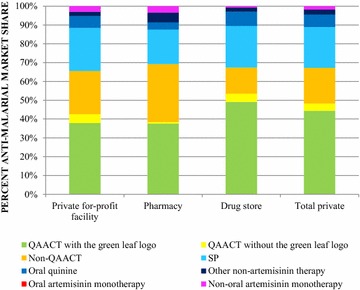
Anti-malarial market share within the private sector

Figure 2 illustrates the market share of different classes of anti-malarials distributed within the private outlets and for the total private sector. Across the private sector, ACT was the most commonly distributed type of anti-malarial (66.1%) and most commonly QAACT (47.5%). Most of the QAACT market share was found in drug stores (52.9%), followed by private for-profit health facilities (41.5%) and pharmacies (38.0%). Across the private sector, SP comprised 21.3% of the anti-malarial market share. No oral artemisinin monotherapy products were found. Non-oral artemisinin therapy made up 1.7% of the overall private market share.

### Availability of anti-malarials and testing

Table 1 illustrates availability of anti-malarials and malaria diagnostic tests among all screened private sector outlets. Among all screened outlets, 93.4% had an anti-malarial in stock on the day of survey. ACT was stocked in 80.7% of the private sector; availability of QAACT was 72.0%. Oral quinine was the most commonly available non-artemisinin therapy (73.2%), followed by SP (47.1%).

**Table 1:** Availability of malaria testing and anti-malarials, among all screened private sector outlets

Availability among all outlets	Private for-profit health facility	Pharmacy	Drug store	Total private sector
	N = 1023 % (CI)	N = 493 % (CI)	N = 1967 % (CI)	N = 3483 % (CI)
*Anti-malarials*				
Any anti-malarial	93.1 (90.4, 95.1)	98.3 (96.4, 99.2)	93.4 (91.3, 95.0)	93.4 (91.8, 94.7)
Any ACT	80.3 (74.2, 85.3)	97.9 (96.1, 98.9)	80.4 (76.9, 83.5)	80.7 (77.3, 83.6)
Any QAACT	69.5 (63.2, 75.1)	94.8 (91.6, 96.8)	72.3 (68.2, 76.0)	72.0 (68.2, 75.4)
SP	48.5 (42.1, 54.8)	82.5 (76.3, 87.3)	45.9 (40.3, 51.7)	47.1 (42.1, 52.1)
Oral quinine	65.9 (60.7, 70.8)	94.1 (89.1, 96.8)	75.0 (70.8, 78.9)	73.2 (69.5, 76.6)
Other non-artemisinin therapy^a^	9.1 (6.2, 13.2)	53.5 (43.1, 63.6)	10.5 (8.4, 13.1)	10.8 (9.1, 12.8)
Injectable quinine	67.1 (60.2, 73.4)	79.5 (70.1, 86.5)	20.0 (16.5, 24.0)	31.9 (28.5, 35.6)
Non oral artemisinin monotherapy	47.7 (42.2, 53.3)	84.0 (77.0, 89.2)	10.9 (8.2, 14.4)	20.6 (17.5, 24.1)
*Malaria diagnostic testing*				
Any diagnostic	70.6 (63.6, 76.7)	51.4 (44.1, 58.6)	20.9 (17.8, 24.4)	32.9 (29.4, 36.5)
RDT	47.4 (41.4, 53.4)	51.4 (44.1, 58.6)	20.1 (17.1, 23.4)	26.8 (23.9, 30.0)
Microscopy	42.0 (35.1, 49.1)	0.6 (0.2, 1.4)	1.2 (0.6, 2.3)	10.6 (8.5, 13.2)
*Readiness for malaria case management (ACT and malaria testing)*				
Quality assured ACT and testing	52.5 (46.2, 58.6)	49.0 (41.9, 56.1)	16.1 (13.6, 19.0)	25.1 (22.2, 28.4)

^a^Other non-artemisinin therapy included: amodiaquine, atovaquone-proguanil, chloroquine, hydroxychloroquine sulphate, mefloquine, primaquine

Malaria blood testing was available in 32.9% of private sector outlets, and highest among private for-profit facilities (70.6%) followed by pharmacies (51.4%) and drug stores (20.9%). Availability of parasitological testing was mainly attributed to RDT, with the exception of private for-profit facilities where both microscopy (42.0%) and RDT (47.4%) were available at similar levels.

ACT and malaria testing was available 25.1% of all private sector outlets. This was highest among private for-profit facilities (52.5%), followed by pharmacies (49.0%) and drug stores (16.1%).

### Price of malaria testing and anti-malarials

In the private sector, the median retail price of a package of adult QA AL was four times more expensive than pediatric QA AL ($1.62 and $0.39 respectively). An AETD of QAACT was also three times more expensive than an AETD of SP ($1.62 and $0.48 respectively) (Table 2). Anti-malarials were typically less expensive in pharmacies and drug stores as compared to private for-profit facilities.

**Table 2:** Median private sector price of malaria testing and anti-malarials

	Private for-profit facility	Pharmacy	Drug store	Private sector total
	Median [IQR]^(N of products)^	Median [IQR]^(N of products)^	Median [IQR]^(N of products)^	Median [IQR]^(N of products)^
Median price of a package of				
Adult QA AL	$1.62 [1.29–2.26] ^(664)^	$1.29 [0.97–1.62] ^(670)^	$1.62 [1.29–1.62] ^(1269)^	$1.62 [1.29–1.94] ^(2603)^
Pediatric QA AL	$0.65 [0.32–0.97] ^(84)^	$0.48 [0.32–0.97] ^(134)^	$0.32 [0.29–0.48] ^(173)^	$0.39 [0.32–0.58] ^(391)^
Median price of tablet AETD				
QAACT	$1.94 [1.29–3.23] ^(1365)^	$1.28 [$0.97–$1.94] ^(1467)^	$1.55 [$0.97–$1.94] ^(2446)^	$1.62 [$1.13–$1.94] ^(4811)^
SP	$0.65 [0.48–0.81] ^(605)^	$0.48 [0.48–0.65] ^(587)^	$0.48 [0.48–0.65] ^(1122)^	$0.48 [0.48–0.65] ^(2314)^
Median price of an ampoule				
Quinine injection	$0.81 [0.48–1.13] ^(691)^	$0.48 [0.32–0.81] ^(453)^	$0.81 [0.32–0.65] ^(307)^	$0.97 [0.48–0.97] ^(1451)^
Artemether injection	$0.97 [0.81–1.62] ^(455)^	$0.54 [0.39–0.65] ^(393)^	$0.81 [0.48–0.97] ^(162)^	$0.97 [0.65–1.29] ^(1010)^
Median price of a microscopy				
Adult	$0.97 [0.65–0.97] ^(409)^	$1.62 [0.97–1.62] ^(8)^	$0.65 [0.48–0.97] ^(19)^	$0.97 [0.65–0.97] ^(436)^
Child under age five	$0.97 [0.65–0.97] ^(410)^	$1.62 [0.97–1.62] ^(8)^	$0.65 [0.48–0.97] ^(18)^	$0.81 [0.65–0.97] ^(436)^
Median price of RDT				
Adult	$0.97 [0.65–0.97] ^(513)^	$0.97 [0.32–0.97] ^(44)^	$0.65 [0.65–0.97] ^(390)^	$0.81 [0.65–0.97] ^(947)^
Child under five	$0.97 [0.65–0.97] ^(514)^	$0.97 [0.32–0.97] ^(44)^	$0.65 [0.65–0.97] ^(391)^	$0.81 [0.65–0.97] ^(949)^

The median retail price for an adult microscopy and RDT was $0.97 and $0.81, respectively. The retail price of an adult and child RDT was $0.81. Malaria diagnosis was least expensive in drug stores compared to other private sector outlet types and the price did not differ by type of test ($0.65).

### Fever case management results

A total of 1266 outlets were identified during the national outlet survey that met the fever case management survey eligibility criteria. Of these eligible outlets, 1146 outlets were visited for the fever case management study. There were 1089 outlets that participated in patient screening and 259 outlets that did not have any eligible patients. Of a total of 830 outlets with complete patient observation and exit interviews, 423 were private for-profit health facilities, 147 were pharmacies and 260 were drug stores.

A total of 9330 patients were screened during the fever case management study. Of these patients, 1273 had complete patient observations and exit interviews. The age of the patient observations and exit interviews ranged from 0 years to over 50 years: 545 patients were between 0 and 4 years; 170 patients were between 5 and 14 years; 503 patients were between 15 and 49 years and 49 patients were over 50 years of age (age data were missing for 6 respondents).

### Fever case management study sample description

Table 3 provides a description of fever patients who were eligible for the fever case management study and had completed both observation and exit interviews. The results show that across the private sector, 74.2% of respondents were patients seeking treatment at outlets as compared to 25.8% of respondents that were seeking treatment on behalf of the patient. 23.1% of respondents had sought treatment elsewhere prior to being interviewed at the facility and this first source of treatment was most commonly from other private facilities (13.8%) rather than the public sector (9.7%). Among all respondents, 8.6% reported receiving a malaria test, 20.5% received a medicine, and 12.5% an anti-malarial at a previous treatment source.

**Table 3:** Description of fever patients, by outlet type

	Private for-profit health facility	Pharmacy	Drug store	All private sector outlets
	N = 630	N = 219	N = 424	N = 1273
Respondent is the patient and present at the outlet/consultation	85.4 (79.5–89.9)	49.4 (36.5–62.3)	65.3 (58.9–71.2)	74.2 (69.5–78.4)
*Prior to the current visit, the percent of all respondents that:*				
Sought treatment from another source	20.8 (16.7–25.7)	37.6 (28.8–47.2)	24.4 (19.3–30.4)	23.1 (19.7–27.0)
Sought treatment from a public sector source	5.9 (3.6–9.4)	18.0 (12.7–25.0)	12.8 (8.9–17.9)	9.7 (7.4–12.6)
Sought treatment from a private sector source	14.9 (11.3–19.3)	19.9 (9.8–36.3)	15.6 (10.3–22.8)	13.8 (10.7–17.7)
Received a malaria blood test from a previous treatment source	6.5 (4.3–9.8)	25.5 (17.3–35.9)	8.5 (5.1–14.0)	8.6 (7.0–10.5)
Received any medicine from a previous treatment source	19.6 (15.6–24.4)	16.7 (10.1–26.4)	22.8 (16.8–30.0)	20.5 (16.8–24.6)
Received an anti-malarial from a previous treatment source	12.4 (9.0–16.8)	7.1 (3.3–14.6)	13.0 (9.8–17.2)	12.5 (9.9–15.8)

### Malaria blood testing

**Fig. 3:**
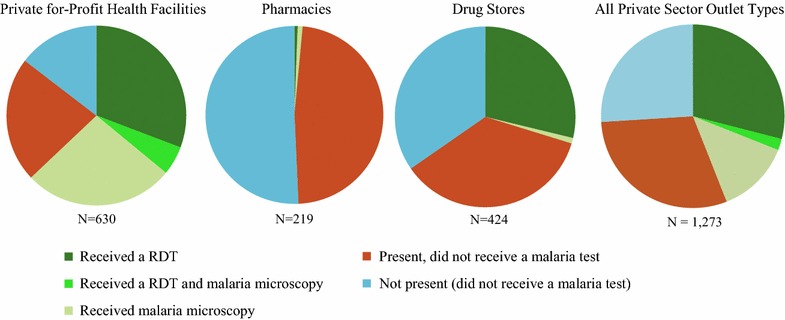
Percentage of respondents who received a malaria blood test, across outlet type

Figure 3 shows the relative distribution of respondents at the outlet according to whether or not they received a malaria diagnostic test, for the private sector and by outlet type. Almost half of all respondents received a malaria test (44.4%), and the most common type of test received was RDT (28.8%). Malaria testing was most common among private for-profit facilities (63.0%), followed by drug stores (29.0%). In 48.5% of pharmacies and 35.6% of drug stores, respondents were patients present at the outlet and did not receive a test.

### Fever treatment by malaria test results

Table 4 illustrates the treatment outcome by malaria test result. Among respondents who tested positive for malaria, 83.0% received an anti-malarial, 60.0% received ACT and 48.5% received QAACT. QAACT was most commonly administered to confirmed positive patients at drug stores (68.4%) (Additional file 2: Table S2). 14.4% of confirmed positive patients received non-artemisinin therapy, mainly quinine injections, tablets or syrups, SP tablets or chloroquine tablets, or an artemisinin monotherapy, which primarily consisted of artemether injections (14.9%). Furthermore, 78.7% of all patients with a positive malaria test received an antipyretic (78.7%), while 42.5% received an antibiotic.

**Table 4:** Fever treatment by malaria test result across all private outlets

	Malaria test positive % (95% CI) N = 266	Malaria test negative % (95% CI) N = 250	Not tested % (95% CI) N = 753
Any anti-malarial	83.0 (74.6–89.0)	14.3 (9.3–21.2)	50.8 (44.4–57.3)
Any ACT	60.0 (53.3–66.3)	10.2 (6.5–15.5)	42.7 (36.4–49.3)
QA ACT	48.5 (41.3–55.7)	6.6 (3.7–11.6)	33.9 (27.7–40.7)
Non-quality assured ACT	12.2 (8.1–18.1)	3.5 (1.3–9.4)	8.9 (5.6–13.7)
Non-artemisinin therapy^a^	14.4 (9.6–21.1)	3.6 (1.3–9.3)	8.4 (5.9–11.8)
Artemisinin monotherapy^b^	14.9 (9.3–23.0)	0.5 (0.1–3.6)	1.1 (0.4–3.3)
Antibiotic	42.5 (34.1–51.3)	54.7 (45.7–63.4)	24.1 (18.9–30.1)
Antipyretic	78.7 (71.4–84.5)	61.2 (50.6–70.9)	64.9 (59.5–70.0)
Patient received a prescription for an anti-malarial, but did not receive an anti-malarial at the outlet (exiting without treatment)	0.2 (0.1, 0.8)	3.3 (0.7, 14.4)	0.2 (<0.1, 1.8)

^a^Primarily quinine injections, tablets, syrups as well as SP tablets and a few chloroquine tablet anti-malarials

^b^Primarily artemether injections

Among patients testing negative for malaria, 14.3% were administered an anti-malarial, 10.2% received an ACT, and 3.6% a non-artemisinin therapy. Over half received an antibiotic (54.7%) and 61.2% received an antipyretic (Table 4).

Among patients who were not tested for malaria, 50.8% were treated with an anti-malarial, 42.7% were treated with ACT and 33.9% QAACT, and 8.4% were given a non-artemisinin therapy. 24.1% were treated with an antibiotic and 64.9% were given an antipyretic (Table 4).

## Discussion

The private sector in Uganda was responsible for most of the anti-malarial distribution, with more than half of anti-malarials administered through this sector in 2015. This is concurrent with other research that most patients seek treatment from the private sector in Uganda [20–23]. Most of the anti-malarials distributed by the private sector were ACT, though one in five anti-malarials distributed were SP, which was notably less expensive than ACT. While in many cases, private providers use available malaria commodities to test fever cases and treat according to test results, gaps exist in appropriate case management. Findings point to recommendations for improving coverage of appropriate malaria case management.

### Role of the private sector in malaria case management

Most of the private sector anti-malarial distribution was through drug stores, comprising one third of the anti-malarial market share followed by private for-profit facilities and pharmacies. Outlets that have been found to play an important role in anti-malarial distribution in other countries such as general retailers and itinerant drug vendors do not provide anti-malarials in Uganda, as evidenced by multiple ACTwatch survey rounds [20, 21]. For example, in 2013, 1241 general retailers were screened for anti-malarials and none were found to be stocking these medicines [21].

Given the importance of drug stores in malaria case management in Uganda, what is known about them? They are authorized to sell over-the-counter medicines and should be licensed by the National Drug Authority. National regulations stipulate that they should be staffed by qualified health providers and administration of medicines should follow national policies. However, in practice it is possible that a substantial proportion of these outlets are not registered [3, 24]. For example, a census of private outlets in three rural eastern districts of Uganda estimated that up to 77.1% of private vendors may be unlicensed [25]. These unlicensed drug stores are described as operating illegally and manned by unqualified staff selling a range of prescription and non-prescription medicines [3]. The presence of these unlicensed drug stores may be particularly common given evidence that the implementation of laws and regulations governing medical practice can be challenging [26].

Strategies to license drugs stores may help to regulate these outlets and allow them to be included as part of the formal health care system. Such efforts may be an important means to improve access to quality malaria case management services. Several strategies in other countries have demonstrated that unlicensed providers have been successfully integrated into the formal health system through training, supervision, business incentives, and accreditation [27]. However, systematic evaluations of these activities have rarely been conducted [28]. While allowing unlicensed drug shops to participate in future programmes may expand the reach of case management services, these should be aligned in the context of Uganda’s national policy and regulatory framework.

### Private sector readiness for appropriate malaria case management

Overall, there was high availability of ACT in the private sector (80.7%). The findings illustrate there was high readiness to administer ACT in the private sector, reflecting increasing availability of ACT over time in Uganda, as evidenced by data from previous outlet surveys [20, 21, 29]. For example, among drug shops surveyed, the percentage of outlets stocking an ACT medicine increased over time from 12.9% in 2009, to 63.5% in 2011 and to 75.1% in 2013. These findings reflect private sector initiatives in Uganda, including the AMFm and CPM, where in 2015, 8.48 million ACT doses had been delivered to private sector First Line Buyers, albeit at reduced levels since a peak of 19.4 million treatment doses in 2013 (personal communication, Global Fund).

While many private sector outlets have QAACT available following on initiatives to improve private sector availability, only a quarter have both QAACT and malaria blood testing available. Access to diagnostic testing has remained low in the private sector, though previous ACTwatch national surveys in Uganda have generally reported an increase over the past decade [20, 21, 29]. In particular, testing availability was moderate to high in pharmacies and private for-profit facilities, but particularly low among drug stores. The findings also reflect population-based studies which have found that less than 15% of febrile children under five received a malaria diagnostic test from the private sector in Uganda [18].

Low private sector availability of malaria blood testing can be partly explained by the national regulatory framework which has only permitted the use of RDT among licensed private drug stores in pilot settings. However, the results from a pilot findings in Uganda are promising and suggest that these outlets can safely and correctly test for malaria with appropriate training, supervision, and record keeping [30, 31]. For example, RDT-positive patients were 5.6% points more likely to buy ACT and 31.4% points more likely to buy other anti-malarials than those not tested at all [32]. While this suggests that a policy in favor of parasitological testing in licensed drug stores may foster increased access and appropriate case management of suspected malaria cases, scaling this up at a national level is not without its challenges. The experience of introducing RDT in the private sector in Cambodia over the last 10 years has shown challenges with RDT supply, as well as determining effective incentives for private providers and patients to use these tests and adhere to their results [33]. In addition, other studies have shown that RDT may result in an increase in prescription rates for antibiotics when RDTs were introduced, particularly in RDT negative cases [34, 35]. This suggests that the introduction of RDTs may also have inadvertent effects on the use of other medicines.

### Private sector anti-malarial distribution

More than half of the anti-malarials distributed in the private sector were ACT (66.1%), and in 2015 ACT with the green leaf logo comprised 43.6% of the market share. Non-artemisinin therapy comprised one-fifth of the market share, with SP most commonly sold/distributed. While there were few differences between outlet types, QAACT market share was highest among drug stores.

The 2015 findings speak to positive improvements in the private sector market share since the implementation of the AMFm program, even while deliveries of ACT with the green leaf logo have been declining since 2013 as funding of co-paid ACT dropped in 2014 and 2015. In 2010, market share for any ACT in Uganda’s private sector was estimated at 5.1% and this increased to 38.5% in 2011. The findings from the most recent survey, reported here, illustrate a further 27.6% point increase of ACT market share. With most of this increase attributed to ACT with the green leaf, this suggests that positive improvements can be associated with the CPM ACT subsidy programme.

Despite widespread distribution of ACT, non-artemisinin therapies continued to be widely available in the private sector and there was still some distribution of these medicines, notably quinine and SP. Findings from the fever case management study also indicate that artemether injections were being administered for confirmed, uncomplicated malaria cases, despite outlets having ACT in stock. While SP should continue to account for a portion of anti-malarial market share because this product is recommended for intermittent preventive treatment in pregnancy (IPTp) [3], the substantial SP market share is cause for concern, and suggests it is being administered for malaria case management, against national (and international) guidelines. This is also supported by other evidence that suggests many of the SP products have packaging and patient instructions indicating its use for uncomplicated malaria for all ages [36].

One reason for the widespread availability and distribution of non-artemisinin therapy may be related to price. The findings from the outlet survey show that QAACT was three times more expensive than SP. QAACT was also more expensive than artemether injection fever case management study. These price barriers could have driven patient demand and the decision by the provider to administer a less expensive treatment option rather than QAACT. In 2010, one of the AMFm supportive interventions included a recommended retail price (RRP) for QAACT, which was $0.47 for an adult dose and $0.12 for a child dose, however consumer awareness raising activities had not yet been implemented by 2015. The results from this study demonstrate that the median price for an adult and child treatment was three times higher than the RRP, at $1.62 and $0.39 respectively. ACT retail prices therefore may not be low enough to achieve optimal uptake, pointing to the need for a further reduction in ACT retail price [6, 11]. Implementation of the planned BCC should be a useful strategy to increase awareness of the recommended retail price for QAACT, and promote demand for this treatment at an affordable price. Such activities could be coupled with a strengthening policies and regulations to curtail the availability and distribution of non-artemisinin therapies for malaria case management in the private sector [37].

### Confirmatory testing in the private sector

The fever case management findings point to sub-optimal private sector case management, illustrating that even in situations where malaria diagnostic testing is available, patients are not routinely tested. The findings demonstrate that among all respondents interviewed, less than half (44%) received a confirmatory test and this was even lower among those visiting drug stores (29%), where most treatment is sought. These findings however are also consistent with other research that has shown irrational treatment decisions by health providers despite availability of diagnostic tests [38–40].

The findings from the fever case management study highlight some of the complexities of malaria diagnosis. For example, one in four of the respondents interviewed were seeking treatment on behalf of someone else who was not present and therefore could not be tested. The findings also illustrate how the patient may have already been managed at a different facility—almost one in four respondents had sought treatment elsewhere prior to attending the outlet where they were interviewed. Similarly, a proportion at the consultation had already been given an anti-malarial at a different facility.

Several other barriers to administering malaria confirmatory testing in the private sector have been documented. This includes whether or not private providers will have an economic incentive to promote and sell RDTs to patients considering the revenue that is generated from anti-malarials [32, 41]. There may also be concern over what to do when a result from a test is negative [33]. From the patient’s perspective, there may also be a financial disincentive to purchase both a test and a medicine. This study found that the price of a malaria test was less than the price of treatment with an ACT for adults. However for children, there was no financial incentive to test before treatment because ACT treatment was cheaper than RDT testing. Although there was an apparent financial incentive to test before treatment with an ACT for adults, the price of testing was still higher than other available non-artemisinin therapies including the popular option, SP. Furthermore, patients seeking malaria treatment may see the price of an RDT test on top of the price they must pay for treatment as an unnecessary cost. In fact, data from a previous study in Uganda illustrated that drug shop customers’ willingness-to-pay threshold for RDTs was considerably lower than the actual RDT price, with many preferring to spend their money on medicines rather than testing [42]. Thus, provider motivation and additional cost to patients for testing remain important challenges to scaling-up diagnostic testing within the private sector.

Several strategies may be useful to overcome these barriers. One important strategy will be to build consumer demand for testing. The findings from the fever case management study show that testing was available in private sector outlets, yet fewer than half of patients received a confirmatory test. Social and behavior change communication that targets patients and provides them with information and education on the importance of confirmatory testing, will be an important means to increase demand for testing. Demand side strategies could also be supported with several supply side interventions to ensure RDTs are affordable and accessible to patients. This may include bundling RDTs and ACT as a single commodity rather than two separate commodities [42], such that if the RDT was positive the patient could then be offered a free or highly subsided ACT. A voucher system for RDTs and ACT may be another fruitful avenue to consider, where a full refund of the RDT is offered for positive patients on the condition that they purchase an ACT [14]. Such private sector supply side strategies could be complemented with aforementioned patient-targeted BCC that promotes RDTs as a necessary and affordable commodity [3]. Of promise is that several strategies have demonstrated successful introduction of RDTs in Uganda’s private sector [12, 13, 43]. Lessons from these studies can pave the way for future scale-up of confirmatory testing and can consider these several options to promote diagnostic testing among all patients.

### Treatment according to test results in the private sector

The findings from the fever case management study show that among private sector outlets that had ACT and diagnostic services available, over 80% of patients who tested positive for malaria received an anti-malarial. While these results are promising, there is still a gap given that one in five patients did not receive an anti-malarial, despite a confirmed positive blood test. It is not clear from the results of this study why these confirmed positive cases did not receive appropriate treatment as these outlets were all stocking ACTs. This could be related to price or patient demand for certain treatments, and/or that patients may have had other options for obtaining treatment elsewhere or at home. While anti-malarial treatment was high among confirmed cases, treatment with an ACT was lower at 60%, highlighting the problem of availability and administration of non-ACTs for treatment of uncomplicated malaria.

The results also illustrate that within the private sector, that patients who are not tested commonly receive treatment with an antipyretic. Treating fever with an antipyretic is appropriate, but patients presenting with fever and are not being tested are a missed opportunity to catch what may be malaria infection and treat it appropriately. Furthermore, half of people not tested were treated with an anti-malarial and confirmatory testing prior to treatment could reduce what may be irrational anti-malarial use.

Administration of an antibiotic was quite high among those with a positive test result (42.5%) and many patients also purchased several medicines, including antipyretics. These findings are consistent with other studies implemented in Uganda's private sector, which have found widespread administration of antibiotics and antipyretics among RDT positive patients [13].

Several studies have suggested that while there is a reduction in anti-malarial treatment after RDT introduction [5–8], anti-malarials are administered despite negative test results. Findings from this study are promising given that anti-malarial and specifically ACT prescription among malaria negative patients was low, and lower than what has been observed in other studies [9–12]. There may be several reasons as to why anti-malarials are still administered despite a negative test result, including concerns of patient safety [44], a mistrust of negative test results [45], or uncertainty as to what to do when faced with a negative result [46].

One way to encourage providers to administer RDT and adhere to test results may include increasing product awareness, both among patients to drive demand, but also for providers to stock and sell RDT. This may require intense BCC activities, as well as provider training. The complexity of messaging is also noted as a key challenge, given there is more than one message and one objective [33]. It has been suggested that messaging should promote the need to be tested prior to treatment, to take a recommended first-line treatment, and if test results are negative to urge the patient not to take an anti-malarial. A key challenge remaining for providers is what to do if the patient tests negative. Simple algorithms may be helpful, but additional provider training and support, as well as a network of referral systems will be necessary to address these obstacles.

## Limitations

The sample for the fever case management study was dependent upon the findings from the concurrently implemented ACTwatch outlet survey, which has previously documented limitations [19, 47]. In the fever case management study, the outlets were identified through the outlet survey census, and only those outlets with ACT and diagnostics were included. Due to the small time lag between the outlet survey and the fever case management study, some previously eligible facilities may have lost eligibility after having stock-outs of either ACT or RDTs. Furthermore, there are notable challenges with observational studies, particularly where multiple data-collecting observers are used as was in the case of this study. It is possible that the observers may have differed in the consistent identification, discrimination and recording of data. It is also likely that social desirability biases may have played a role in affecting provider behavior. The presence of fieldworkers observing provider practices may have led to some change in their behaviour.

## Conclusion

The private sector is a common source of anti-malarial treatment for people in Uganda. While many private sector outlets have QAACT available following on from initiatives to improve private sector availability, only one-quarter have both QAACT and testing available. Results show that in many instances, private providers who stock ACT and malaria blood testing often use available commodities to appropriately manage patients. However, gaps persist in ensuring all fever patients receive a confirmatory test and QAACT. There is need to further promote confirmatory testing and ACT among patients and private sector providers, as well as discourage the use of non-artemisinin therapies and inappropriate use of injectable artemisinin monotherapies for cases of uncomplicated malaria.

## Additional files

**Additional file 1.** Detailed outlet survey sample description.

**Additional file 2.** Fever treatment by malaria test result and outlet type.

## Abbreviations

ACT: artemisinin-based combination therapy
AL: artemether lumefantrine
AETD: adult equivalent treatment dose
AMFm: affordable medicines facility for malaria
BCC: behaviour change communications
CHW: community health worker
CPM: co-payment mechanism
EMA: European Medicines Agency
IQR: interquartile range
IPTp: intermittent preventive treatment in pregnancy
mRTD: malaria rapid diagnostic test
PACE: Programme for Accessible Communication and Education
QAACT: quality assured artemisinin combination therapy
SP: sulfadoxine pyrimethamine
WHO: World Health Organization
