# Supplementary material for: Private sector role, readiness and performance for malaria case management in Uganda, 2015
Source: Malar J. 2017 May 25;16:219. doi: 10.1186/s12936-017-1824-x (PMC5445348; doi:10.1186/s12936-017-1824-x)
Supplement: Supplementary file 1 — Additional file 1. Detailed outlet survey sample description. [file 12936_2017_1824_MOESM1_ESM.docx]

Additional File Table S1: Detailed Outlet Survey Sample Description

|  | Public  Health  Facility | CHW | Private not for-profit facility | Private  for-profit  facility | Pharmacy | Drug store | ALL  Outlets |
| --- | --- | --- | --- | --- | --- | --- | --- |
| **Number of outlets screened** | 282 | 5,618 | 55 | 1,023 | 493 | 1,967 | 9,438 |
| Census | 178 | 5,618 | 55 | 1,023 | 161 | 1,967 | 9,002 |
| Booster | 104 | 0 | 0 | 0 | 332 | 0 | 436 |
| **Number of outlets eligible and interviewed** | 281 | 1,012 | 54 | 980 | 476 | 1,921 | 4,724 |
| Census | 178 | 1,012 | 54 | 980 | 157 | 1,921 | 4,302 |
| Booster | 103 | 0 | 0 | 0 | 319 | 0 | 422 |
| **Number of outlets eligible but not interviewed** | 0 | 3 | 0 | 19 | 15 | 19 | 56 |
| Census | 0 | 3 | 0 | 19 | 2 | 19 | 43 |
| Booster | 0 | 0 | 0 | 0 | 13 | 0 | 13 |
| **Number of interviewed outlets with at least one anti-malarial in stock on the day of the survey** | 275 | 719 | 53 | 956 | 476 | 1,849 | 4,328 |
| Census | 173 | 719 | 53 | 956 | 157 | 1,849 | 3,907 |
| Booster | 102 | 0 | 0 | 0 | 319 | 0 | 421 |
| **Number of interviewed outlets with at least one anti-malarial in stock on the day of the survey or at least one anti-malarial reportedly in stock in the previous 3 months** | 280 | 900 | 54 | 967 | 476 | 1,921 | 4,598 |
| Census | 177 | 900 | 54 | 967 | 157 | 1,921 | 4,176 |
| Booster | 103 | 0 | 0 | 0 | 319 | 0 | 422 |
| **Number of interviewed outlets that provide malaria blood testing, but do not stock anti-malarials** | 1 | 112 | 0 | 13 | 0 | 0 | 126 |
| Census | 1 | 112 | 0 | 13 | 0 | 0 | 126 |
| Booster | 0 | 0 | 0 | 0 | 0 | 0 | 0 |
| **Number of interviewed outlets that reported distributing anti-malarials in the week prior to the survey** | 177 | 357 | 30 | 633 | 302 | 1,306 | 2,805 |
| Census | 110 | 357 | 30 | 633 | 95 | 1,306 | 2,531 |
| Booster | 67 | 0 | 0 | 0 | 207 | 0 | 274 |
| **Number of interviewed outlets that reported providing/distributing a malaria diagnostic test in the week prior to the survey** | 133 | 211 | 37 | 428 | 17 | 94 | 920 |
| Census | 74 | 211 | 37 | 428 | 5 | 94 | 849 |
| Booster | 59 | 0 | 0 | 0 | 12 | 0 | 71 |
