# Supplementary material for: Private sector role, readiness and performance for malaria case management in Uganda, 2015
Source: Malar J. 2017 May 25;16:219. doi: 10.1186/s12936-017-1824-x (PMC5445348; doi:10.1186/s12936-017-1824-x)
Supplement: Supplementary file 2 — Additional file 2. Fever treatment by malaria test result and outlet type. [file 12936_2017_1824_MOESM2_ESM.docx]

Additional File Table S2: Fever treatment by malaria test result and outlet type

|  |  | **Malaria test positive^##^**  **% (95% CI)** | **Malaria test negative^###^**  **% (95% CI)** | **Not tested^####^**  **%**  **(95% CI)** |
| --- | --- | --- | --- | --- |
| Any anti-malarial | Facility | 74.1  (62.4-83.1) | 11.5  (6.3-20.3) | 43.0  (34.0-52.3) |
|  | Pharmacy | 100.0  (-) | 28.7  (5.0-75.4) | 55.3  (42.0-68.0) |
|  | Drug Store | 97.2  (89.6-99.3) | 22.2  (11.6-38.4) | 54.3  (46.5-62.0) |
| Any ACT | Facility | 49.0  (41.5-56.5) | 7.5  (4.2-13.1) | 37.0  (27.3-47.7) |
|  | Pharmacy | 88.1  (35.8-99.0) | 14.8  (1.4-68.8) | 43.0  (32.3-54.4) |
|  | Drug Store | 77.5  (64.3-86.8) | 17.9  (8.6-33.8) | 45.5  (38.0-53.2) |
| QA ACT | Facility | 35.9  (28.7-43.8) | 5.7  (2.8-11.3) | 31.7  (22.9-42.1) |
|  | Pharmacy | 75.6  (22.5-97.1) | 14.8  (1.4-68.8) | 24.8  (15.6-37.0) |
|  | Drug Store | 68.4  (55.4-79.0) | 9.3  (3.5-22.6) | 35.6  (27.5-44.7) |
| Non-QA ACT | Facility | 13.1  (8.4-19.8) | 1.8  (0.7-4.4) | 5.2  (2.6-10.2) |
|  | Pharmacy | 12.4  (1.1-65.4) | 0.0  - | 18.2  (10.1-30.7) |
|  | Drug Store | 10.8  (5.1-21.5) | 8.6  (1.8-32.4) | 9.9  (5.3-17.7) |
| Non-artemisinin therapy | Facility | 12.2  (7.0-20.6) | 3.3  (1.1-9.9) | 7.5  (4.2-13.2) |
|  | Pharmacy | 11.9  (1.0-64.2) | 0.0  - | 10.6  (6.0-17.9) |
|  | Drug Store | 18.0  (10.3-29.6) | 4.3  (0.6-24.9) | 8.6  (5.7-12.9) |
| Artemisinin monotherapy | Facility | 19.7  (12.6-29.4) | 0.7  (0.1-4.9) | 0.1  (<0.1-0.4) |
|  | Pharmacy | 87.6  (34.6-98.9) | 13.8  (1.7-59.3) | 1.8  (0.5-6.2) |
|  | Drug Store | 7.0  (2.6-17.5) | 0.0  - | 1.6  (0.5-5.1) |
| Antibiotic | Facility | 46.9  (37.7-56.4) | 51.1  (41.3-60.8) | 23.1  (15.0-33.8) |
|  | Pharmacy | 87.6  (34.6-98.9) | 27.7  (2.7-84.1) | 25.9  (17.6-36.3) |
|  | Drug Store | 35.3  (22.4-50.7) | 65.4  (40.4-84.0) | 24.4  (17.7-32.5) |
| Antipyretic | Facility | 72.6  (61.2-81.6) | 57.8  (45.2-69.4) | 61.2  (51.2-70.4) |
|  | Pharmacy | 100.0  - | 42.5  (5.6-90.2) | 56.1  (40.9-70.2) |
|  | Drug Store | 88.4  (76.2-94.7) | 71.5  (54.5-84.0) | 67.4  (59.9-74.1) |
| Patient received a prescription for an anti-malarial | Facility | 0.4  (0.1, 1.2) | 4.4  (0.9, 18.3) | 0.0  - |
|  | Pharmacy | 0.0  - | 0.0  - | 0.0  - |
|  | Drug Store | 0.0  - | 0.0  - | 0.4  (0.1, 2.7) |

^##^ Private for-profit health facilities, N=188; Pharmacies, N=3; Drug stores, N=75

^###^ Private for-profit health facilities, N=196; Pharmacies, N=4; Drug stores, N=50

^####^ Private for-profit health facilities, N=244; Pharmacies, N=212; Drug stores, N=297
